# Growth and Characterization of* M*-Plane GaN Thin Films Grown on *γ*-LiAlO_2_ (100) Substrates

**DOI:** 10.1155/2017/2362084

**Published:** 2017-10-18

**Authors:** Yu-Chiao Lin, Ikai Lo, Hui-Chun Shih, Mitch M. C. Chou, D. M. Schaadt

**Affiliations:** ^1^Department of Physics, Department of Materials and Optoelectronic Science, Center for Nanoscience and Nanotechnology, National Sun Yat-sen University, Kaohsiung 80424, Taiwan; ^2^Institute of Energy Research and Physical Technologies, Clausthal University of Technology and Energy Research Center of Lower Saxony, Clausthal-Zellerfeld, Germany

## Abstract

*M*-plane GaN thin films were grown on LiAlO_2_ substrates under different N/Ga flux ratios by plasma-assisted molecular beam epitaxy. An anisotropic growth of *M*-plane GaN was demonstrated against the N/Ga flux ratio. As the N/Ga flux ratio decreased by increasing Ga flux, the GaN surface trended to a flat morphology with stripes along [11$\overset{-}{2}$0]. According to high-resolution X-ray diffraction analysis, Li_5_GaO_4_ was observed on the interface between GaN and LiAlO_2_ substrate. The formation of Li_5_GaO_4_ would influence the surface morphology and crystal quality.

## 1. Introduction

The wurtzite GaN and its ternary compounds were investigated widely in the last few decades because of their outstanding physical stable properties and the potential for high performance optoelectronic devices [1, 2]. Due to the strong piezoelectric and spontaneous polarization fields along the *c*-axis of GaN which were attributed to the dipole and stress asymmetry, the polar *c*-plane GaN-based quantum wells (QWs) suffered from quantum-confined stark effect (QCSE) in the growth direction. As a result, the band structure of *c*-plane GaN would be modified that further reduced the overlap of electron-hole wave functions, which were disadvantages for its optoelectronic performance [3, 4]. In order to eliminate the effect of QCSE in the growth direction, a possible solution is to grow nonpolar (e.g., *M*-plane and **a**-plane) GaN-based QWs [5].

The *γ*-LiAlO_2_ (LAO) substrate, which is tetragonal crystal structure and belongs to the space group P4_1_2_1_2, is an ideal substrate for *M*-plane GaN epitaxial growth. Its lattice constants are identified as **a**_LAO_ = **b**_LAO_ = 0.5169 nm and *c*_LAO_ = 0.6268 nm. The LAO substrate shows a greatly small lattice mismatch with *M*-plane GaN. The lattice mismatches between them are −1.7% and −0.3% in [11$\overset{-}{2}$0] and [0001] directions of GaN, respectively [6–9]. However, the LAO substrate is hydrolytic and thermally less stable [10]. It is difficult to grow high quality *M*-plane GaN on LAO substrates by general growth methods such as metal-organic chemical vapor deposition (MOCVD) or hydride vapor phase epitaxy (HVPE), which were processing at more than 1000°C. In the previous studies, we have grown *M*-plane GaN on LAO and misoriented LAO substrates at relatively low growth temperature growth by plasma-assisted molecular beam epitaxy (PAMBE) and have found a large anisotropic growth mechanism and strain within the *M*-plane GaN films [11, 12]. In this study, we grew a series of *M*-plane GaN thin films (about 80 nm) by PAMBE and investigated their in-plane anisotropic properties.

## 2. Materials and Methods

Five samples of *M*-plane GaN thin film, labeled as samples A, B, C, D, and E, were grown on LAO substrates by PAMBE system with standard effusion cell for Ga evaporation (99.9995% purity) and ultra-high pure nitrogen gas (99.9999% purity) supplied in a radio-frequency plasma source (Veeco model GEN 930). The 1 × 1 cm^2^ LAO substrates were cut from polished 2 inch wafer, and the LAO crystal ingot was fabricated by using the traditional Czochralski pulling technique. Before mounting on a holder, the LAO substrates were degreased with acetone, isopropanol, and phosphoric acid (H_3_PO_4_ : H_2_O = 1 : 30) in an ultrasonic bath for five minutes sequentially and deionized water for a few seconds and then dried with nitrogen gas immediately. Before epitaxial growth, a thermal treatment, out-gassed at 850°C for 10 minutes, was introduced to the LAO substrates and then the Ga wetting layer was performed for 5 minutes at 800°C in the MBE growth chamber. The GaN were grown for 30 minutes at 800°C under different N/Ga flux ratios. The N/Ga flux ratios of samples A, B, C, D, and E were 60.0, 54.5, 52.2, 50.0, and 45.8 (with the Ga flux were 1.00 × 10^−7^, 1.10 × 10^−7^, 1.15 × 10^−7^, 1.20 × 10^−7^, and 1.30 × 10^−7^ torr), respectively. The N/Ga flux ratios were evaluated from the beam equivalent pressure of N source against that of Ga source. The in situ reflection high-energy electron diffraction (RHEED) was used to characterize the growth of GaN thin films. The surface morphology was obtained by scanning electron microscope (SEM) (JEOL JSM-6330TF). The structural properties and crystalline preferred orientations were characterized by high-resolution X-ray diffraction (XRD, Bede D1) using a SIEMENS D5000 X-ray diffractometer with a Cu anode and field emission transmission electron microscope (FE-TEM) (Phillips, model Tecnai F-20) with an electron voltage of 200 kV. The cross-sectional TEM specimens were prepared by focus ion beam (FIB) (Seiko Inc., model SII-3050). The optical properties of the samples were analyzed by polar-dependence photoluminescence (PL) (Horiba, Lab RAM HR Evolution) spectra which was measured using a continuous wave He–Cd laser (325 nm).

## 3. Results and Discussion

**Figure 1:**
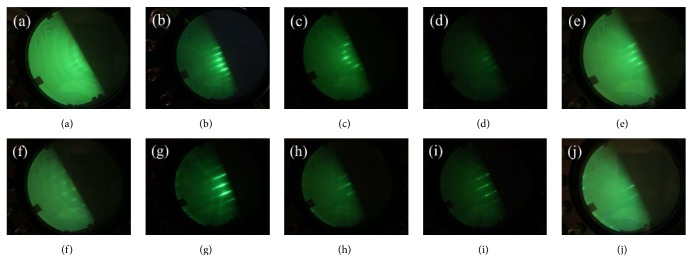
RHEED patterns of samples A, B, C, D, and E for [11$\overset{-}{2}$0] azimuth (a)–(e) and for [0001] azimuth (f)–(j).

Figure 1 shows the RHEED patterns for GaN [11$\overset{-}{2}$0] and GaN [0001] azimuth of the samples. The RHEED patterns of sample A (N/Ga = 60.0) show streaky patterns for [11$\overset{-}{2}$0] azimuth in Figure 1(a) and spotty patterns for [0001] azimuth in Figure 1(f). It indicated the in-plane anisotropic growth: 2D-like growth along [11$\overset{-}{2}$0] and 3D-like growth along [0001] of GaN. In addition, the ringed patterns were observed for both [11$\overset{-}{2}$0] and [0001] azimuth of sample A. It revealed that the GaN was a mixed structure, including polycrystalline and single crystalline. As the N/Ga flux ratio was decreased to 54.5 and 52.2 for samples B and C, the RHEED patterns of samples B and C exhibit a bright streaky pattern in Figures 1(b), 1(c), 1(g), and 1(h), indicating the growth mechanism of GaN which trended to 2D-like growth under these growth conditions. While the ratio kept decreasing, the smeared streaky patterns were presented for samples D and E, with the N/Ga ratios of 50.0 and 45.8, as shown in Figures 1(d), 1(e), 1(i), and 1(j), respectively. Since the GaN films were grown under such low N/Ga flux ratios, the smeared streaky patterns were induced by excess Ga atoms under the Ga-rich condition. It was noted in Figure 1(i) that the intensity of the RHEED pattern for [0001] azimuth of sample D shows a modulated streaky pattern, which revealed that the growth condition of sample D (especially in [0001] direction) was under an intermediate growth mode between 2D-like growth and 3D-like growth.

**Figure 2:**
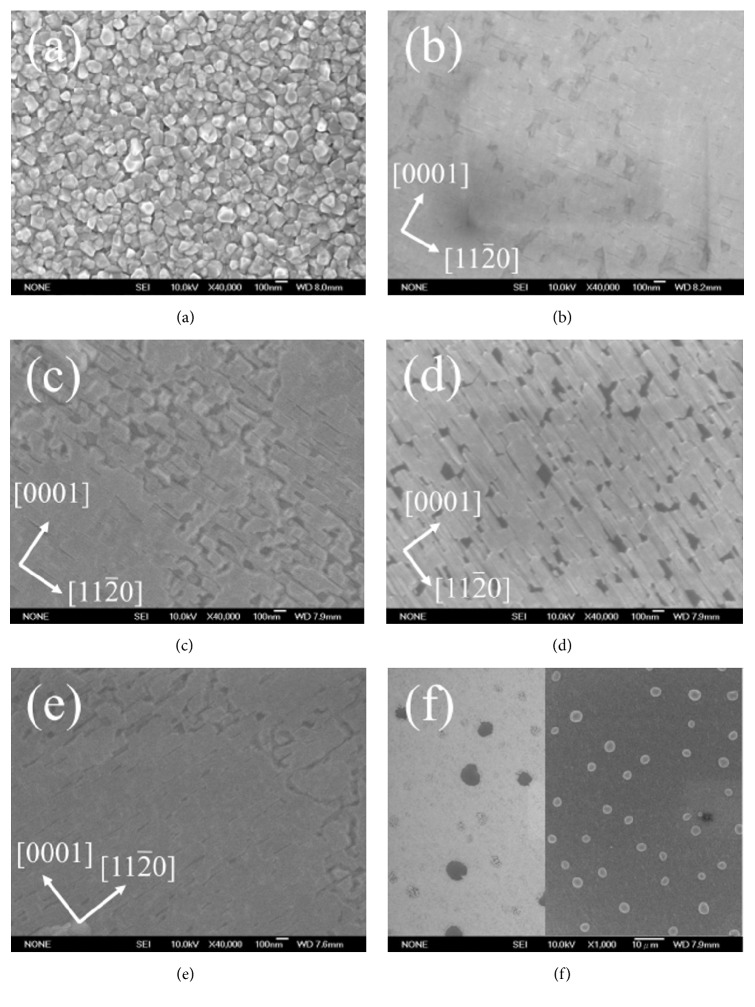
SEM images of samples A, B, C, D, and E (a)–(e); the scale bar is 100 nm. SEM image of samples D (left) and E (right) (f); the scale bar is 10 *μ*m.

The anisotropic growth mechanism could be attributed to the anisotropic lattice mismatch and thermal expansion mismatch between *M*-plane GaN and LAO substrates. Consequently, this anisotropic surface diffusion behavior will be the longer growth steps in the [11$\overset{-}{2}$0] direction due to the lower diffusion barrier and the shorter steps bunching in the [0001] direction due to higher diffusion barrier [13]. According to the SEM image in Figure 2(a), sample A shows the morphology of polycrystalline with small grain size (about 100 nm). Sample A was grown under the highest N/Ga ratio (N/Ga = 60.0). Under such high N/Ga flux ratio (low Ga flux), the LAO substrate would be damaged by the N_2_ plasma during growth so that GaN could not be epitaxially grown on the LAO substrate although we had grown Ga wetting layer before grown GaN. As the N/Ga flux ratio was decreased to 54.5 and 52.2 for sample B and C, the surface of both samples B and C exhibits a flat morphology with stripes parallel to [11$\overset{-}{2}$0], as shown in Figures 2(b) and 2(c), due to the lower diffusion barrier. We also observed rectangular pits and cracks in the central area of the SEM images for samples B and C. Those pits and cracks could be attributed to the GaN grown on the interstices of Ga droplets. When the Ga wetting layer was grown on the LAO substrate for 5 minutes, it formed numerous Ga droplets which did not cover the surface of LAO substrate completely. Therefore, those uncovered areas would be damaged by the N_2_ plasma, leading to a rough morphology with pits and cracks. For the sample D (N/Ga = 50.0), the surface shows a rough morphology with longer step edges in [11$\overset{-}{2}$0] direction and shorter step edges in [0001] direction, as shown in Figure 2(d). Since the N/Ga flux ratio decreased to 50.0, the growth condition trended to a Ga-rich regime, resulting in a lot of Ga droplets induced by excess Ga atoms during growth.  Figure 2(f) shows the SEM image, which was scanned under wider field of view, of sample D (left side) and sample E (right side). It could be observed that there were a great amount of Ga droplets on the top. However, as the N/Ga flux ratio kept decreasing for sample E (N/Ga = 45.8), the surface presented a flat morphology with stripes parallel to [11$\overset{-}{2}$0] as shown in Figure 2(e), which was similar to the morphology of samples B and C. The pits and cracks were observed on the upper right side of SEM image, as well.

**Figure 3:**
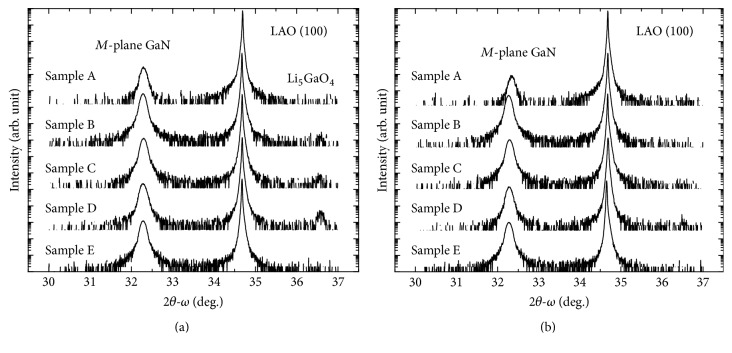
XRD 2*θ*-*ω* scan diagrams for [11$\overset{-}{2}$0] azimuth (a) and [0001] azimuth (b).

**Figure 4:**
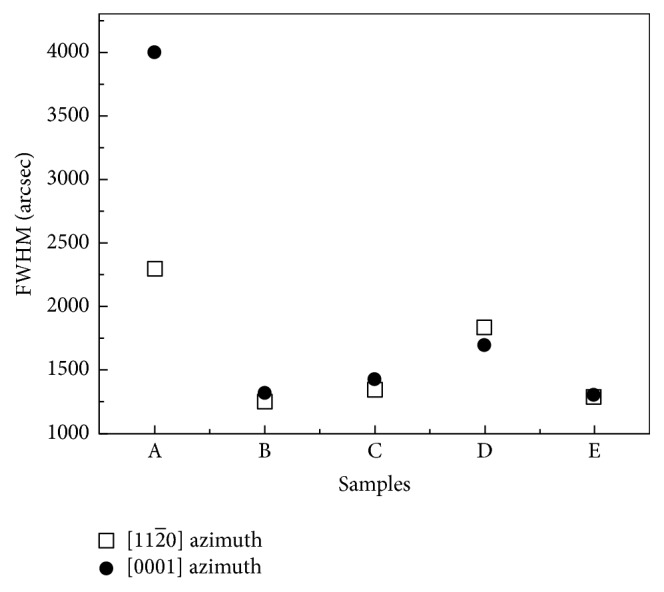
Rocking curve FWHM values of samples A, B, C, D, and E for [11$\overset{-}{2}$0] azimuth (open square) and [0001] azimuth (solid circle).

To determine the crystallographic orientation, the samples were characterized by XRD for different azimuth.  Figure 3 shows the XRD 2*θ*-*ω* scan diagrams for (a) GaN [11$\overset{-}{2}$0] azimuth and (b) GaN [0001] azimuth of the samples. In the 2*θ*-*ω* scan diagrams, the highest peak at 2*θ* = 34.68° is corresponding to the diffraction of LAO (100) and a lower peak is corresponding to the diffraction of *M*-plane GaN (1$\overset{-}{1}$00). The diffraction peaks of GaN (1$\overset{-}{1}$00) of samples A, B, C, D, and E were 32.2918°, 32.2809°, 32.2943°, 32.2827°, and 32.2838° for [11$\overset{-}{2}$0] azimuth and 32.3420°, 32.2692°, 32.2911°, 32.2825°, and 32.2763° for [0001] azimuth, respectively. The shift of the peak position from the theoretical *M*-plane GaN diffraction angle (2*θ*_Theo_ = 32.38°) could be attributed to the in-plane compressive strain. It should be noticed that there was a weak peak at 2*θ* = 36.58° in the 2*θ*-*ω* scan diagrams for [11$\overset{-}{2}$0] azimuth of samples B, C, and D, as shown in Figure 3(a). This peak is corresponding to Li_5_GaO_4_ (123) or Li_5_GaO_4_ (312). Under the growth conditions of samples B, C, and D, a part of LAO would be damaged by N_2_ plasma with the Ga wetting layer, yielding to the formation of Li_5_GaO_4_ during the initial of GaN growth. It was found that the Li_5_GaO_4_ peak was the strongest in sample D, which leads to a rough morphology as shown with SEM image in Figure 2(d). When the N/Ga flux ratio kept decreasing for sample E, the growth condition (with N/Ga flux ratio = 45.8) favored the epitaxial growth of GaN to suppress the formation of Li_5_GaO_4_ during the growth. As a result, the peak of Li_5_GaO_4_ became stronger when the N/Ga flux ratio decreased (samples B, C, and D) and vanished as the N/Ga ratio decreased to 45.8 (sample E). The XRD *ω*-scan rocking curve of *M*-plane GaN was taken for [11$\overset{-}{2}$0] and [0001] azimuth as well. The full width at half maximum values (FWHM) of rocking curve reflected the quality of samples with overall defect density and anisotropic mosaicity [14]. The FWHM of *M*-plane GaN for [11$\overset{-}{2}$0] and [0001] azimuth were shown in Figure 4. Sample A shows wider rocking curves (FWHM for [11$\overset{-}{2}$0] and [0001] azimuth were 2296 arcsec and 4402 arcsec). The wider rocking curve represents a poorer crystal quality. As the N/Ga flux ratio decreased, the FWHM of sample B for [11$\overset{-}{2}$0] and [0001] azimuth were reduced to 1243 arcsec and 1320 arcsec, respectively. However, the FWHM for [11$\overset{-}{2}$0] and [0001] azimuth of sample C (sample D) were 1339 (1830) arcsec and 1284 (1360) arcsec, respectively. The poorer crystal quality of samples C and D could be attributed to the formation of Li_5_GaO_4_. Since Li_5_GaO_4_ could not be observed in sample E, the FWMH for [11$\overset{-}{2}$0] and [0001] azimuth were reduced to 1284 arcsec and 1306 arcsec, which indicated that the crystal quality was improved when Li_5_GaO_4_ was absent.

**Figure 5:**
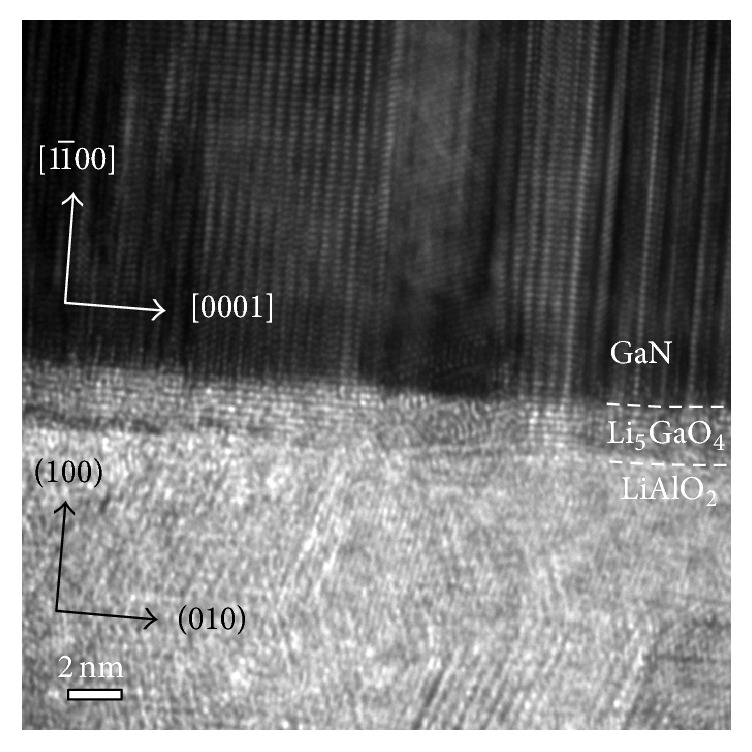
Cross-sectional TEM image of sample D.

The high-resolution cross-sectional TEM image of sample D was taken along [11$\overset{-}{2}$0], as shown in Figure 5. A clear interlayer (about 2 nm) was observed between *M*-plane GaN and LAO substrate. The interlayer could be attributed to the self-assemble Li_5_GaO_4_. If the *M*-plane GaN samples were grown under N-rich condition, LAO substrates reacted under irradiation of N_2_ plasma and decomposed into the binary compounds (i.e., Al_2_O_3_ or Li_2_O). Generally, LiXO_2_ would transform into Li_5_XO_4_ (X = Al, Ga) under the high-temperature condition or irradiation damage [15, 16]. In our case, those binary compounds reacted with Ga atoms, provided by Ga wetting layer and leading to the formation of Li_5_GaO_4_. As the *M*-plane GaN was grown under Ga-rich growth condition by increasing the Ga flux (sample E), the irradiation damage was prevented resulting in the absence of Li_5_GaO_4_.

**Figure 6:**
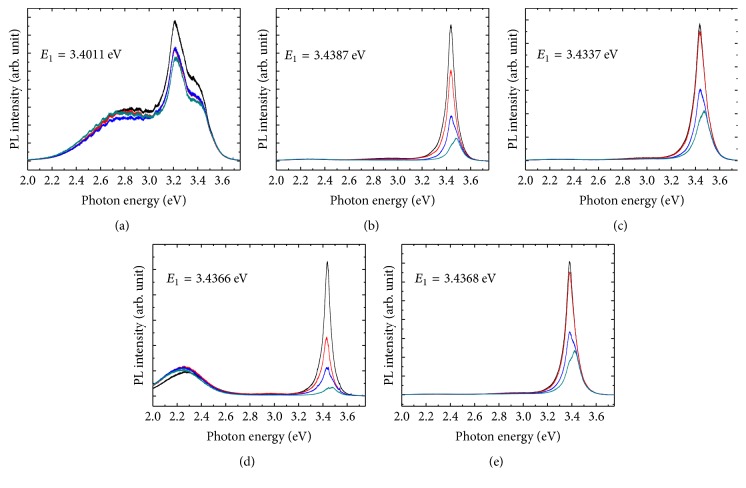
Polar-dependence PL spectra of sample A (a), B (b), C (c), D (d), and E (e). The polarization angles were 90° (black), 60° (red), 30° (blue), and 0° (dark green), respectively.

Due to the in-plane strain within the *M*-plane GaN films, the maxima of valence bands at the Brillouin-zone center (Γ-point) are split into three energy levels, that is, heavy hole (HH), light hole (LH), and spin-orbital crystal-field split-off hole (SCH) [17]. Under the anisotropic compressive in-plane strain condition, the energy levels of interband transition were modified in the order of HH, SCH, and LH versus conduction band, of which corresponding emission energies are *E*_1_, *E*_2_, and *E*_3_, respectively [18]. The polar-dependence PL spectra taken at room temperature were shown in Figure 6. As the polarization angles decreased from 90° to 0°, the emission transformed from *E*_1_ to *E*_2_. Because sample A was a polycrystalline structure, the PL spectra of sample A contain a defect-related emission (2.6~2.8 eV) and the zinc-blende (about 3.2 eV) GaN, wurtzite GaN emission (including an *M*-plane *E*_1_ emission peak at 3.4011 eV). The *E*_1_ emission of samples B, C, D, and E were 3.4387 eV, 3.4337 eV, 3.4366 eV, and 3.4368 eV, respectively. The slightly shift of *E*_1_ emission could be attributed to the in-plane compressive strain. The polar-dependence PL spectra support the analyses of XRD measurements and SEM observations.

## 4. Conclusion

We have grown five *M*-plane GaN thin films on the LAO substrates under different N/Ga flux ratios by PAMBE. Because of the anisotropic diffusion mechanism of adatoms, RHEED patterns show a streaky patterns for [11$\overset{-}{2}$0] azimuth. For [0001] azimuth, it shows streaky, spotty, and modulated streaky patterns for different samples. Under the extreme N-rich growth condition, LAO substrates would be damaged by N_2_ plasma, leading to a polycrystalline structure. The crystal quality could be improved and the surface trended to a smoother morphology by decreasing the N/Ga flux ratio. As the N/Ga flux ratio kept decreasing, the crystal quality became poorer and the surface trended to a rough morphology. The rough morphology and poorer crystal quality could be attributed to the formation of Li_5_GaO_4_ which was observed by the XRD 2*θ*-*ω* scan diagram for [11$\overset{-}{2}$0] azimuth. The self-assembled Li_5_GaO_4_ was formed if the *M*-plane GaN films were grown under the intermediate growth condition between N-rich and Ga-rich, and it could be suppressed by the growing of *M*-plane GaN under Ga-rich condition.
